# Nanosecond pulsed platelet‐rich plasma (nsPRP) improves mechanical and electrical cardiac function following myocardial reperfusion injury

**DOI:** 10.14814/phy2.12710

**Published:** 2016-02-23

**Authors:** Barbara Hargrave, Frency Varghese, Nektarios Barabutis, John Catravas, Christian Zemlin

**Affiliations:** ^1^ Department of Medical Diagnostics and Translational ScienceOld Dominion UniversityNorfolkVirginia; ^2^ Frank Reidy Center for BioelectricsOld Dominion UniversityNorfolkVirginia; ^3^ Department of Electrical EngineeringOld Dominion UniversityNorfolkVirginia

**Keywords:** Heat‐shock proteins, ischemic–reperfusion injury, mitochondria, platelet‐rich plasma, spare respiratory capacity

## Abstract

Ischemia and reperfusion (I/R) of the heart is associated with biochemical and ionic changes that result in cardiac contractile and electrical dysfunction. In rabbits, platelet‐rich plasma activated using nanosecond pulsed electric fields (nsPRP) has been shown to improve left ventricular pumping. Here, we demonstrate that nsPRP causes a similar improvement in mouse left ventricular function. We also show that nsPRP injection recovers electrical activity even before reperfusion begins. To uncover the mechanism of nsPRP action, we studied whether the enhanced left ventricular function in nsPRP rabbit and mouse hearts was associated with increased expression of heat‐shock proteins and altered mitochondrial function under conditions of oxidative stress. Mouse hearts underwent 30 min of global ischemia and 1 h of reperfusion in situ. Rabbit hearts underwent 30 min of ischemia in vivo and were reperfused for 14 days. Hearts treated with nsPRP expressed significantly higher levels of Hsp27 and Hsp70 compared to hearts treated with vehicle. Also, pretreatment of cultured H9c2 cells with nsPRP significantly enhanced the “spare respiratory capacity (SRC)” also referred to as “respiratory reserve capacity” and ATP production in response to the uncoupler FCCP. These results suggest a cardioprotective effect of nsPRP on the ischemic heart during reperfusion.

## Introduction

The term ‘’ischemic–reperfusion injury” describes several types of myocardial dysfunction inclusive of, but not limited to: (1) myocardial stunning, which is reversible and is manifested by mechanical dysfunction after ischemia and the restoration of blood flow (Heyndrickx et al. 1975) (generally, there is no evidence of cell necrosis) and (2) no‐flow ischemia, which occurs when there is microvascular obstruction presenting as tissue compression, endothelial and myocyte swelling, and neutrophil infiltration, resulting in the inability to completely perfuse the ischemic area. Cardiac necrosis is present along with ventricular arrhythmias (Kloner et al. 1980). Ischemia and reperfusion (I/R) of the heart is associated with biochemical and ionic changes that result in cardiac contractile and electrical dysfunction (Carmeliet 1999). While there are several effective therapeutic methods for re‐establishing blood flow to the ischemic heart (Tiefenbrunn and Sobel 1992; Rogers et al. 1994), no such therapeutic intervention exists to protect and treat damaged cardiac myocytes secondary to I/R.

There are various forms of metabolic stress that predispose I/R cardiomyocytes to damage. One of the best described mechanisms of I/R injury is oxidative stress, resulting from the formation of reactive oxygen species (ROS). Elevated ROS levels can cause oxidative damage to the cells they encounter (Miller and Cheung 2015). During early reperfusion the mitochondrial electron chain, activated neutrophils, cardiac myocytes, and endothelial cells are all sources of ROS production (Barandier et al. 1999; Park and Lucchesi 1999; Hoffman et al. 2004). Under physiologic conditions, ROS produced from these sources are detoxified by endogenous antioxidants (Hoffman et al. 2004). However, under conditions of I/R, when the increase in ROS levels exceeds the capacity of the endogenous scavenging mechanisms, cell damage can occur (Hoffman et al. 2004).

During cardiac ischemia there is an insufficient availability of oxygen for mitochondria to carry out oxidative metabolic reactions. As a result, there is an approximate 65% decrease in ATP levels after 15 min of ischemia and a 90% decrease after 40 min (Reimer and Jennings 1986).

We have previously demonstrated that platelet‐rich plasma (PRP) prepared using nanosecond pulsed electric fields (nsPRP) to activate the platelets, reduces ROS levels in cultured H9c2 (*Rattus norvegicus*) and HUVEC (human umbilical vein endothelial) cells stimulated with H_2_O_2_ (Hargrave 2014). We have also reported that nsPRP contains significant concentrations of the antioxidants catalase (CAT) and superoxide dismutase (SOD) (Hargrave 2014), something that we did not observe in PRP activated with the conventional bovine thrombin as the platelet activator (Hargrave 2014). These observations provide evidence in support of an effect of nsPRP on ROS levels and on CAT and SOD, and suggest that the method used for the preparation of PRP may be important. More importantly, these data led us to propose that lower ROS levels may contribute to the enhanced left ventricular function that we (Hargrave and Francis 2012) and others (Cheng et al. 2012) previously observed in vivo and in situ in the rabbit and rat I/R heart models. Here, we show that nsPRP enhances LV function in mice and that it also partially restores electrical activity (in rabbit). We previously reported that ischemic–reperfused rabbit hearts treated with nsPRP had improved LV function under dobutamine stress and that infarct size was smaller (Hargrave and Francis 2012). To investigate the mechanisms by which nsPRP improves LV function, we measured the expression of the cardioprotective heat‐shock proteins Hsp 27 and Hsp70 in vivo in rabbit tissue and the mitochondrial spare respiratory capacity in cultured H9c2 cells.

## Materials and Methods

Animal experimentation was approved by the Institutional Animal Care and Use Committee at Old Dominion University and was conducted in accordance with the Guide to the Care and Use of Laboratory Animals (NIH publication). Nine adult female mice and 16 adult female New Zealand white (NZW) rabbits were studied.

### Ischemia–reperfusion in vivo rabbit heart model

The surgical preparation for the in vivo rabbit I/R model has been described by Hargrave and Francis (2012). Briefly, eight New Zealand white rabbits (Harlan, Inc., Frederick, Maryland) were sedated with a combination of acepromazine (2 mg/kg) and ketamine (25 mg/kg) and intubated using a pediatric endotracheal tube size 3. Once the endotracheal tube was in place, general anesthesia was induced using isoflurane in oxygen (5%–5 L/min for 1 min then 1.5%–1.5 L/min for the duration of the procedure). The endotracheal tube was attached to a Harvard respirator and the animal ventilated at 50 breaths/minute using a nonrebreathing circuit.

The left thoracic area was shaved and cleansed with 70% ethanol and chlorhex‐Q scrub. Once a surgical plane of anesthesia was established, the animal was surgically prepared for the creation of ischemia and reperfusion by performing a left thoracotomy and pericardiotomy. To make the heart ischemic, the distal branch of the left anterior descending artery was transiently occluded by placing a 5‐0‐prolene suture around the distal segment of the vessel. The coronary vessel was occluded for 30 min by placing tension on the suture. Ten minutes before release of the occlusion and the start of reperfusion, 200 *μ*L of nsPRP supernatant or 200 *μ*L saline was injected directly into the myocardium of the left ventricle at the level of the infarcted tissue using a sterile 25‐G 1/2‐inch needle. After all injections were made, the thoracic cavity was closed and care was taken to remove excess air from the cavity to prevent the development of a pneumothorax. An angiocath (16 G) was used as a chest tube to remove air from the chest cavity. The chest tube was then removed. Once the chest was closed and the incision sutured, the isoflurane was stopped and the animal was allowed to breathe room air. The endotracheal tube was removed when there were signs that the animal was breathing comfortably without the ventilator. All animals were returned to their housing facility for 14 days.

#### Measurement of left ventricle mechanical function in vivo during dobutamine stress test

Fourteen days after coronary occlusion and reperfusion, general anesthesia was again induced with isoflurane and oxygen as described previously (Hargrave and Francis 2012). A midline incision was made over the trachea and the left carotid artery exposed. A Millar Probe Catheter‐Model SPR 524 (Millar Instruments, Houston, Texas) was gently passed into the left ventricle via the carotid artery for the measurement of left ventricular pressures, heart rate, and positive and negative dp/dt. A 25‐G catheter was placed into the marginal ear vein for the administration of dobutamine (5, 10, and 20 *μ*g/kg/min), which was used as a stressor. The dobutamine (Sigma‐Aldrich, St. Louis, Missouri) dose was increased every 3 min until the maximum dose was achieved. Data were collected for 60 min after the maximal dobutamine dose was reached.

### Mouse Langendorff heart preparation

Nine female adult mice (Swiss Webster, Charles Rivers Laboratory) were studied. A surgical plane of anesthesia was induced by allowing the animal to breathe isoflurane and oxygen. As we described previously (Hargrave and Francis 2012), a thoracic incision was made and the mouse heart was quickly removed and placed into a modified Tyrode's solution chilled to 0–4°C and mounted with the following modifications. The heart was mounted onto a 22‐G 1/2‐inch flanged stainless steel cannula. A Millar catheter (Millar Instruments, Houston, Texas) which was attached to a pressure transducer was inserted into the left ventricle. Left ventricular positive dP/dt was recorded every 10 sec through a polyvinyl catheter using a COBE CDX III transducer and Micro‐Med 100 Blood Pressure Analyzer (Louisville, Kentucky) software. The preparation was allowed to beat spontaneously and permitted to equilibrate for 10 min prior to the initiation of the experimental protocol. nsPRP supernatant or vehicle (0.9% NaCl) injections were made into the myocardium of the left ventricle using a 27‐G 1/2‐inch needle. In each case, the volume of the injectate was 50 *μ*L.

#### H9c2 cell culture

H9c2 (*Rattus norvegicus*; cardiac myoblast) cells (CRL‐1446, ATCC; Manassas, Virginia), were grown in sterile 75 cm^2^ flasks at 37°C and 5% atmospheric CO_2_ as described previously (Hargrave 2014). Briefly, cell culture media consisted of 15 mL Dulbecco's modified Eagle's medium^™^ (Mediatech, Inc., Manassas, Virginia) containing 10% fetal bovine serum (FBS, Atlanta Biologicals, Inc., Lawrenceville, Georgia). Once the cells reached 85–95% confluency, approximately 1.05 × 10^5^ cells/mL were transferred onto a sterile multiwell culture plate (Corning) and treated with 200 *μ*L of ns‐PEF‐activated PRP in 1 mL of cell culture media [1.05 mg/75 cm^2^ flask] or cell culture media only. The cells were then returned to the incubator for 24 h. For the measurement of ROS, cells were loaded with 20 *μ*mol/L CM‐H_2_DCFDA as per the manufacturer's instructions (Invitrogen–Molecular Probes, Eugene, Oregon). ROS formation was measured using flow cytometry (BD FACSAria Cell Sorter, San Jose, California).

#### Nanosecond pulse electric field (nsPEF) and the pulse generator

Nanosecond pulse electric fields are ultrashort pulsed electric fields, typically with high field strength that affects intracellular as well as extracellular membranous structures and functions (Schoenbach et al. 2007; Zhang et al. 2008). Nanosecond pulses convey intense, high power, but low‐energy electric fields. During platelet activation, nsPEFs charge the platelet membrane, and create pores without inducing permanent damage (Schoenbach et al. 2007; Zhang et al. 2008). The 300 nsec pulse generator used for these studies has been described previously (Schoenbach et al. 2007; Zhang et al. 2008). The electrical pulses were applied to PRP in sterile aluminum electroporation cuvettes having an electrode gap of 0.4 cm and an area of 1 cm^2^. The shape and amplitude of the pulse voltage was monitored using a 500‐MHz oscilloscope. For the generation of nsPRP, one milliliter of PRP in the presence of 10 mmol/L CaCl_2_ was exposed to 5 pulses at an electric field of 30 kV/cm.

### Preparation of nsPRP supernatant

We prepared the nsPRP supernatant as described previously by Hargrave (2014). Briefly, the platelet concentrate was exposed to 5 pulses at an electric field of 30 kV/cm.

#### Transfection of H9c2 cells in culture with siRNA Hsp 70

H9c2 cells were exposed to either an Hsp70 siRNA or an irrelevant control siRNA, and then pretreated with nsPRP or vehicle for 24 h followed by stimulation with H_2_O_2_. The cells were loaded with CM‐H2DCFDA, a ROS dye indicator, and analyzed using flow cytometry.

#### Western blot analysis

We evaluated the effect of nsPRP on the expression of the Hsp70 and Hsp27 in the left ventricular rabbit tissue using western blot analysis. In this in vivo study, the hearts were made ischemic by placing a suture around the distal 1/3 of the left anterior descending (LAD) coronary artery and occluding blood flow for 30 min. The suture was removed and the tissue allowed to reperfuse. The chest was closed and the animals maintained for 14 days. On day 14, the LV was separated from the rest of the heart and frozen on dry ice and stored at −80°C until analysis. On the day of analysis, the tissue was homogenized and the proteins were isolated using RIPA buffer. The protein concentration was determined by the BCA protein method according to the manufacturer's instructions (Thermo Fisher Scientific, Rockford, Illinois). Protein‐matched samples (40 *μ*g per lane) were separated by electrophoresis through 12% sodium dodecyl sulfate (SDS‐PAGE) Tris HCl gels. Wet transfer was used to transfer the proteins onto nitrocellulose membranes. The membranes were incubated for 1 h at room temperature in 5% nonfat dry milk in Tris‐buffered saline–0.1% (vol/vol) Tween 20 (blocking buffer). The membranes were then incubated at 4°C overnight with the appropriate Hsp27 (#2402), pHsp27 (#2401), Hsp70 (#4872) antibodies from Cell Signaling (Danvers, Massachusetts), and Hsp90 antibody (#624088) from BD Transductions Laboratories (San Jose, California). All the antibodies were diluted 1/1000 (vol/vol) in blocking buffer. The next day and after a 1 h incubation of the membranes with the corresponding secondary antibodies (LI‐COR, Lincoln, Nebraska) protein expression was visualized using an Odyssey 9120 Infrared Imaging System (LI‐COR, Lincoln, Nebraska). Hsp90 was used as the loading control.

#### Densitometry/statistical analysis

Image J software (National Institutes of Health) was used to perform densitometry of immunoblots. All data are expressed as mean values ± SE (standard error of mean). Student's *t* test was performed to determine statistically significant differences between the two groups. A value of *P *<* *0.05 was considered significant. GraphPad Prism 4 (version 4.03, Graph Pad Software) was used for data analysis; *n* represents the number of experimental repeats.

#### Optical mapping Langendorff perfusion

The optical mapping setup is shown in Figure 5A and has been previously described by Zemlin et al. (2008). Briefly, the heart was surgically removed as described previously and placed in the main chamber of a life support system. It was perfused retrogradely through the aorta with Tyrode solution (in mmol: NaCl 130, KCl 4.0, CaCl_2_ 1.8, MgCl_2_ 1.0, NaHCO_3_ 24, NaH_2_PO_4_ 1.2, glucose 5.6), at a pressure of 60–80 mmHg, maintained by adjusting the perfusion flow rate. The solution was bubbled with a gas mixture of 95% oxygen and 5% CO_2_ and the pH maintained at 7.4. The main chamber was further superfused with the same oxygenized Tyrode solution to keep the temperature of the heart and the surrounding bath at 37 ± 0.5°C. A pressure transducer (TD1000, Transducer Direct) was connected very close to the aorta and resultant pressure signal was continuously sampled by a data acquisition board, displayed, and recorded. Optical mapping of transmembrane voltage (V_m_) was performed. In each case, the motion of the heart was interrupted by adding 5–10 mmol/L blebbistatin (Grinvald et al. 1982; Fedorov et al. 2007), and the heart was loaded with a fluorescent dye. The heart was illuminated with diffused laser light of appropriate wavelength. The fluorescent light was filtered through an appropriate emission filter and the passing light collected by a fast, highly sensitive CCD camera (Little Joe, SciMeasure, Decatur, Georgia).

#### V_m_ mapping

The voltage‐sensitive dye RH237 (Grinvald et al. 1982) (Invitrogen, Carlsbad, California) was used to image transmembrane voltage. RH‐237 was excited with a green laser (SDL‐532‐1000T, up to 1000 mW at 532 nm, Shanghai Dream Lasers) and emits in the green to red. A long‐pass filter with cutoff wavelength 610 nm was used to record RH‐237 emission.

#### Bioenergetics analysis

Oxygen consumption rates (OCR) were measured in real time under basal conditions and in response to indicated mitochondrial inhibitors using the XF^e^24 Seahorse Analyzer (Seahorse Bioscience, North Billerica, MA). The cells were washed with XF assay medium containing glucose (0.45 g) sodium pyruvate (100 mmol/L) and l‐glutamine 4 mmol/L (pH adjusted to 7.4) as per the manufacturer's instructions and incubated for 1 h in a 37°C air incubator. An XF^e^24 plate containing 1 × 10^5^ H9c2 cells/well was transferred to a temperature controlled (37°C) Seahorse (extracellular flux) analyzer and subjected to an equilibration period. The assay cycle comprised a 3‐min mix, 2‐min wait, and 3‐min measure period. After three basal assay cycles, oligomycin (2 *μ*mol/L) was added by automatic pneumatic injection to inhibit the ATP synthase and thus approximate the proportion of respiration used to drive ATP synthesis. After three more cycles, carbonyl cyanide‐4 (trifluoromethoxy) phenylhydrazone (FCCP) (2 *μ*mol/L) was added in the same way to stimulate maximal respiration in mitochondria by uncoupling ATP synthesis from electron transport. After another three cycles, rotenone plus antimycin A (0.5 *μ*mol/L) was added to determine the nonmitochondrial respiratory rate, which was subtracted from all other rates. Coupling efficiency was calculated as the oligomycin‐sensitive fraction of mitochondrial respiratory activity. The experimental cells were treated with nsPRP (1:4 nsPRP to cell culture media) for 24 h prior to analysis.

### Statistical analysis

We used the unpaired two‐tailed Student's *t* tests to determine whether two sets of measurements were significantly different.

## Results

### Mouse Langendorff heart

**Figure 1:**
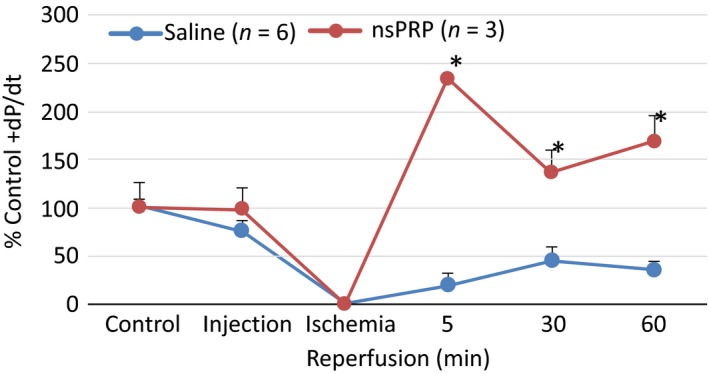
nsPRP improves mouse LV function following I/R**.** Mouse Langendorff‐perfused hearts were treated with 50 µL saline (vehicle) or nsPRP injected into the myocardium 5 min before 30 min of global ischemia, followed by 60 min of reperfusion. The data have been normalized to a baseline of 100%. The baseline dp/dt for the nsPRP hearts was 2526.3 and 2489.2 mmHg for the vehicle‐treated hearts. All data are expressed as means ± SEM. *P <* *0.01 from corresponding vehicle group time point. Data are representative of six vehicle‐ and three nsPRP‐treated hearts.

We have previously reported that nsPRP enhances left ventricular function in the rabbit heart both in vivo and in situ (Hargrave and Francis 2012; Hargrave 2014). To establish that this response was not restricted to rabbit, we analyzed the response of the mouse heart to global ischemia and reperfusion in the presence or absence of nsPRP treatment (Fig. 1). Five minutes after reperfusion was started, +dp/dt was significantly enhanced in nsPRP‐treated hearts and remained so throughout the 60‐min period of reperfusion. These observations are consistent with our findings in the I/R rabbit model (Hargrave and Francis 2012; Hargrave 2014).

### Analysis of electrical activity in Langendorff rabbit hearts using optical mapping

Electrical activity diminished as ischemia progressed and was reduced to noise level in all hearts (*n *=* *7) after 20 min. At this time point, we injected nsPRP in some hearts (n = 4) and saline in other hearts (n = 2), while one heart received no injection. Only after 30 min of ischemia was reperfusion initiated.

**Figure 2:**
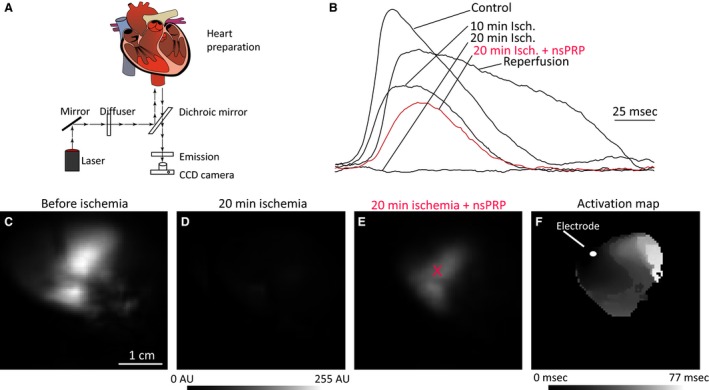
Effect of PRP on electrical activity. (A) Optical mapping setup (see text for details). (B) Action potentials close to the injection site at various time points during ischemia and reperfusion. Action potential amplitude maps before ischemia (C), after 20 min of ischemia (D), and 1 min after injection of nsPRP (E, injection occurred after 20 min of ischemia, injection site is marked with a red “x”). (F) Activation map for tissue that has recovered electrical activation after nsPRP injection (1 min after injection). Tissue was stimulated, the electrode position is marked.

Surprisingly, we observed that nsPRP injection prompted a recovery of electrical activity even before reperfusion. Figure 2B shows the optically measured electrical signals at the nsPRP injection site at various time points in a representative example. While electrical activity had completely ceased after 20 min, it recovered to an amplitude of about 50% of the control level within 1 min of nsPRP injection (nsPRP was administered after 20 min of ischemia). After 30 min, reperfusion was started, and 30 more minutes later, the electrical activity reached and amplitude of about 75% of the control level. Figure 2C–E shows amplitude maps obtained at different time points. The control amplitude map (Panel C, taken before the onset of ischemia) exhibits strong fluorescence; the spatial heterogeneities result from nonuniformities in staining, illumination, and tissue structure. Panel D shows the amplitude map after 20 min of ischemia; at this point, virtually all electrical activity has stopped. Panel E shows a substantial recovery of electrical activity just 1 min later, after nsPRP has been injected. The region in which we observe recovery surrounds the injection site, so we conclude that recovery occurred in all parts of the heart that received a sufficient amount of nsPRP. Panel F shows the activation map of the tissue that has recovered after nsPRP injection. The map clearly shows that one contiguous wave moves over the whole recovered region. While conduction is expectedly slow (~ 10 cm/sec), there are no conduction blocks.

In four hearts in which we injected nsPRP, we always observed recovery of electrical activity, ranging from 43 to 74% of the control amplitude. Activity generally recovered further during reperfusion to 74–89% of the control amplitude (except for one heart that deteriorated dramatically during the last ten minutes of ischemia and did not recover during reperfusion). In contrast, two hearts in which we injected saline and the heart that did not receive any injection showed no recovery. The recovery after reperfusion was to 83–85% of the control amplitude, comparable to the nsPRP‐injected hearts.

### Expression of Hsps 27 and 70 and transfection of H9c2 cells in culture with siRNA Hsp 70

**Figure 3:**
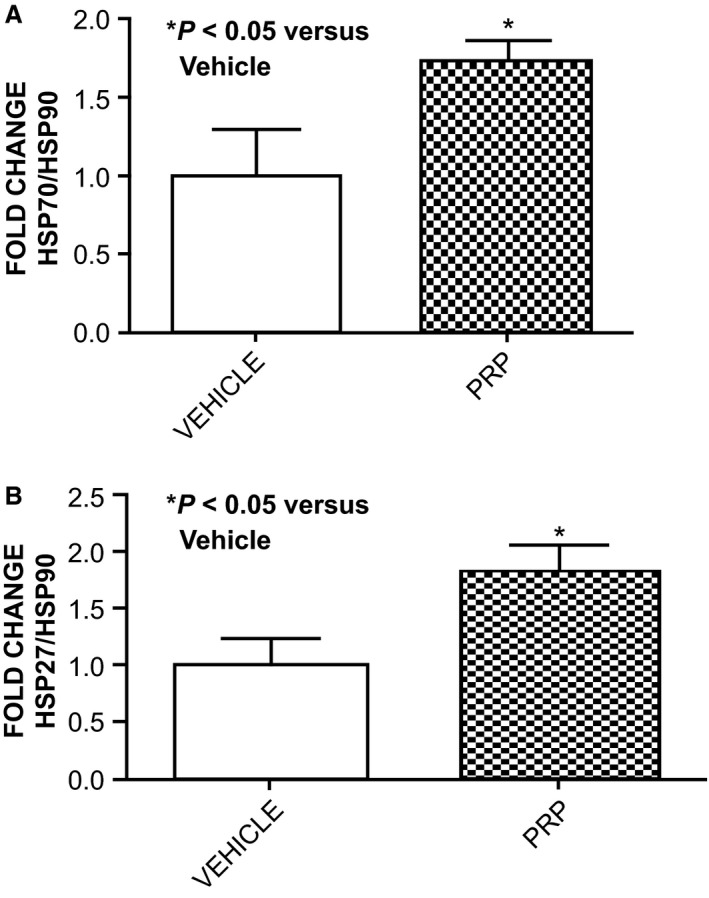
nsPRP increases Hsp70 and Hsp27 myocardial expression following I/R. Expression of Hsp70 and Hsp27 in rabbit LV tissue 14 days after I/R and treatment with nsPRP or vehicle are shown. All data are expressed as means ± SEM. Data are representative of four experimental repeats.

We analyzed the expression of Hsp 27 and Hsp70 in rabbit hearts exposed to nsPRP in vivo. Hearts treated with nsPRP expressed significantly higher levels of Hsp 27 and Hsp 70 (Fig. 3) compared to hearts treated with vehicle.

**Figure 4:**
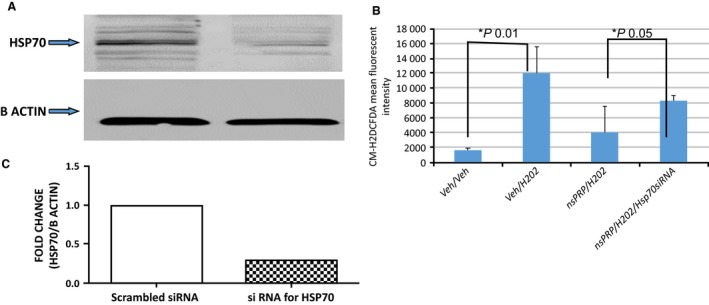
(A) siRNA knock‐down of Hsp70 in H9c2 cells in culture. There was a 70% decrease in Hsp70 expression in cells transfected with Hsp70 siRNA. (B) nsPRP attenuate H_2_O_2_‐induced ROS production via an Hsp70‐sensitive mechanism. H_2_O_2_ (44 mmol/L, 10 min) induced a powerful release of ROS in H9c2 cells. Cells pretreated (24 h) with nsPRP (0.5 µL of nsPRP in 3 mL cell culture media) and stimulated with H_2_O_2_ produced less ROS than cells not receiving nsPRP. However, this effect was partially reversed in cells transfected with Hsp70 siRNA. All data are expressed as means ± SEM. Data are representative of four experimental repeats.

To determine if the decrease in ROS we previously reported (Hargrave and Francis 2012) was related to the changes we observed in the heat‐shock protein expression, we transfected H9c2 cells with siRNA Hsp 70. We observed a 70% decrease in Hsp70 (Fig. 4A). Importantly, cells transfected with siRNA against Hsp70 produced significantly more ROS than cells transfected with the irrelevant siRNA, even in the presence of nsPRP (Fig. 4B), suggesting that nsPRP may require Hsp70 for inhibiting ROS formation. These findings demonstrate an association between nsPRP‐induced HSP expression and ROS inhibition.

### Bioenergetics capacity in cultured H9c2 cells in culture in the presence or absence of nsPRP

**Figure 5:**
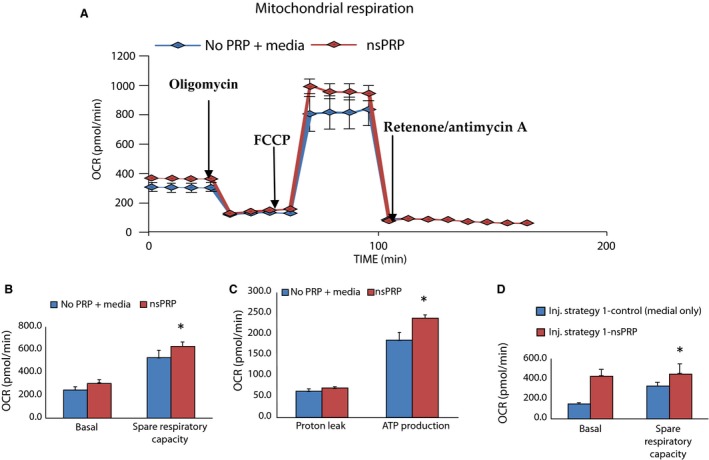
(A) The mitochondrial profile of cultured H9c2 cells pretreated with nsPRP. The cells were exposed sequentially to oligomyosin, which inhibits ATP synthase (complex V), and the decrease in OCR correlates to the mitochondrial respiration associated with cellular ATP production. FCCP (carbonyl cyanide‐4) is an uncoupling agent that collapses the proton gradient leading to an uninhibited flow of electrons through the ETC. ETC and oxygen is maximally consumed by complex IV, and rotenone IV. Rotenone and antimycin A inhibits complexes I and III, respectively, and shuts down mitochondrial respiration. Spare respiratory capacity is a measure of the cell's ability to respond to increased energy demand and is calculated by subtracting the maximal OCR from the baseline OCR. nsPRP increases the spare respiratory capacity. *P < 0.001 for FCCP injection (B) and ATP production (C). Mito stress analysis was performed in the presence or absence of nsPRP pretreatment for 24 h, in H9c2 cells. Control cells were exposed to cell culture media (control) only. (D) Spare respiratory capacity in treated and control cells exposed to 8 mmol/L H_2_O_2_ and treated as previously described. Data were normalized to the number of cells/well. Data are representative of three independent experiments.

The signature profile of H9c2 mitochondria is shown in Figure 5A. There was no significant difference in the basal oxygen consumption rates (OCR) between the nsPRP‐treated and vehicle‐treated cells (Fig. 5A and B). Qualitative and quantitative spare respiratory capacity (Fig. 5A and B, respectively) and ATP production (Fig. 5C) were significantly greater in the nsPRP‐treated cells than in the cells exposed to vehicle. In separate experiments (Fig. 5D), we exposed H9c2 cells pretreated with nsPRP for 24 h to H_2_O_2_ (8 mmol/L) and observed that spare respiratory capacity still trended higher than that observed in the control cells. OCR increased in an effort to confront the increased energy demands. The pretreatment of cultured H9c2 cells with nsPRP significantly enhanced the “spare respiratory capacity (SRC)” also referred to as “respiratory reserve capacity” and ATP production in response to the uncoupler FCCP (Fig. 5A). When oligomycin was added we observed a brief spike in the extracellular acidification rate (a measure of glycolysis) under our experimental conditions (data not shown), suggesting that the XF system was operating properly.

## Discussion

Ischemic–reperfusion injury is a clinically significant event that is associated with morbidity and mortality (Won et al. 2010). Therefore, it is critical to develop therapeutic strategies that improve not only acute survival but the long‐term quality of life. nsPRP is a safe, nonchemical, autologous therapeutic agent that appears to be effective in supporting left ventricular function in the ischemic–reperfused heart. In this study, we have investigated mechanisms by which nsPRP is capable of enhancing left ventricular function in the animal heart exposed to ischemia and reperfusion.

The use of PRP is an approach that is emerging as a potential direct method to deliver angiogenic and growth factors to the ischemic myocardium. Platelet‐rich plasma has provided useful and interesting results (Won et al. 2010; Cheng et al. 2012; Hargrave and Francis 2012; Hargrave 2014). However, the reproducibility of these results has been the subject of controversy and skepticism. In addition, several problems have so far dampened enthusiasm for the use of PRP in the cardiovascular system of patients: (1) injecting a platelet‐rich plasma that contains red blood cells into the heart may lead to thrombi that can cause stroke or myocardial infarction, (2) thrombin can cause serious adverse effects in patients and can interfere with the efficacy of PRP, and (3) lack of a mechanism explaining the mode of action of PRP. We recently modified the production of PRP in two important ways. First, we found a new way to activate the platelets to release the growth factors stored in their *α* granules in the preparation of PRP by using nanosecond pulsed electric fields (nsPEFs). Platelets activated using this methodology appear to preserve the endogenously present antioxidants (Hargrave and Francis 2012; Hargrave 2014), something that we did not observe when the platelets were activated with bovine thrombin. Platelets activated by nsPEFs are similarly effective functionally to thrombin‐activated platelets and in some cases are more effective (Hargrave 2014), and are without the adverse effects of thrombin. Second, we have shown that by using the supernatant of nsPRP, we can eliminate cells (the causes for thrombi) and still retain effectiveness (Hargrave 2014).

There are several mechanisms of myocardial reperfusion injury. However, two major mechanisms are oxidative stress (primarily oxygen‐free radicals) and endothelial dysfunction. Although it is unlikely that any one single pathologic mechanism causes I/R injury, the sum of multiple events, occurring simultaneously, increases the chance of irreversible damage to the myocardium. For example, ischemia and reperfusion are associated with increased ROS levels, vascular dysfunction, and mitochondrial damage. Intriguingly, preservation of these antioxidants was not observed in the PRP prepared using bovine thrombin as the platelet activator (Hargrave 2014). Here, we show that the partial inhibition of ROS by nsPRP may be mediated via the heat‐shock proteins (Fig. 3B) and by stabilization of the mitochondrial membrane.

The reduction of ROS levels in nsPRP‐treated cells was not only observed in H9c2 cells but also in HUVEC cells (Hargrave 2014), and suggests that lower ROS levels may protect the vascular endothelium as well as cardiomyocytes from extensive damage under I/R conditions, thereby preserving function possibly via regulation of the heat‐shock proteins.

The enhanced left ventricular function we observed in the globally ischemic/reperfused mouse Langendorff preparation is consistent with what we (Hargrave and Francis 2012; Hargrave 2014) and others (Cheng et al. 2012) have reported. However, how nsPRP enhances LV function is unclear. In this study, we demonstrated that nsPRP treatment of I/R heart tissue in the rabbit is associated with increased expression of two cardioprotective proteins, Hsp 27 and Hsp 70. Hsp 27 has been shown to be antiapoptotic and cardioprotective for myocytes under stressful conditions such as I/R (Blunt et al. 2007). Blunt et al. have shown that H_2_O_2_ activation of Hsp 27 protects desmin from calpain proteolysis in rat ventricular myocytes (Blunt et al. 2007). Although Hsp 27 is a potent protein, its low transduction efficiency and instability, and short half‐life in the body limits its in vivo application (Christina et al. 2012). Hsp 70 overexpression of in H9c2 cells increase the resistance to an ischemic stress. In 1996, Dillman et al. showed that occlusion of the LAD in dog hearts induced the expression of Hsp 70. We have shown that the transient ligation of the LAD followed by 14 days of reperfusion in rabbits is associated with increased expression of Hsps 27 and 70 (Dillmann et al. 1986). Although it seems likely that expression of the Hsps may have occurred earlier than the 14‐day time period, we only have data supporting a significant increase in expression on day 14 of the experiment. These data suggest one possible explanation for the enhanced LV function. Following ischemic events such as CABG surgery, the stress‐inducible heat‐shock protein Hsp70 has been detected in myocardial cells (Braunwald and Kloner 1982; Dillmann et al. 1986; Chen and Knowlton 2011). In addition, studies show that CABG surgery and ischemic–reperfusion injury are associated with increased production of ROS (Murphy 2004; Rosca and Hoppel 2010), with an ensuing decreased antioxidant defense. The upregulation of expression of Hsp27 and Hsp70 in the presence of nsPRP may, at least in part, explain the enhanced LV function during I/R and may suggest that a reduction in ROS levels (mediated via Hsp) coupled with the presence of the antioxidants CAT and SOD in the tissue, prevent ROS from reaching levels that overwhelm the endogenous free radical scavenging systems thereby reducing ROS damage to myocytes and mitochondria. Our findings are consistent with the ischemia and reperfusion associated with CABG surgery when stress‐inducible Hsp70 has been detected in myocardial cells (Murphy 2004; Rosca and Hoppel 2010).

In this study, we also demonstrate that the spare respiratory capacity in H9c2 cells in culture increases in the cells treated with nsPRP. Mitochondrial dysfunction occurs in ischemic heart disease (Ferrari and Williams 1986; Murphy 2004; Brady et al. 2006). Under conditions such as I/R, the heart requires additional cellular energy in response to stress or increased workload. If the reserve respiratory capacity of the ventricular myocytes is not sufficient to provide the required ATP, cell death can occur. nsPRP may function to protect this spare respiratory capacity (SRC), and thereby reduce cell death. nsPRP‐treated H9c2 cells have an enhanced SRC compared to cells treated with vehicle. SRC is a measure of the cell's ability to respond to increased energy demand. It is the extra capacity available in myocytes to produce energy in response to increased stress caused by I/R and as such is involved in cell survival. Heart cells, like other cell types operate at a basal level that only requires a part of their total bioenergetics capability. The difference between ATP produced by oxidative phosphorylation at basal and that at maximal activity is called the SRC.

We measured the bioenergetic capacity of H9c2 cells in culture to determine whether they use the SRC in response to stress caused by ROS. We observed that intact H9c2 had a substantial capacity to respond to increasing energy demand under basal conditions and in the presence of increased ROS. However, the reason for this response is unclear. It is possible that cell biosynthesis may have increased in the cells treated with nsPRP. We feel that this is unlikely since the data were normalized to the cell number per well with the cell number/well remaining fairly consistent between nsPRP‐ and vehicle‐treated cells. Ferrari et al. reported that although there was a slight reduction in the number of mitochondria isolated from I/R hearts, there were only small functional changes observed, suggesting the presence of a “reserve function in mitochondria extracted from severely damaged cardiac tissue (Ferrari and Williams 1986). Whatever the mechanism, our studies clearly point to enhancement of the SRC in nsPRP‐treated cells in vitro, and enhanced LV work function in the rabbit in vivo and the Langendorff mouse and rabbit hearts in situ following I/R. This increased cardiac capacity may make the heart less vulnerable to bioenergetic exhaustion and thereby decrease the risk of inducing cell death and organ failure.

The ischemic myocardium is prone to increased irritability which can increase the incidence of arrhythmias or sudden cardiac death (Brady et al. 2006). The role that the mitochondria exert in controlling cardiac excitation contraction coupling, excitability, and arrhythmias is not very clear. nsPRP appears to reduce mitochondrial depolarization in both H9c2 and HUVEC cells in culture (Hargrave 2014) suggesting that it in some way stabilizes myocyte and mitochondrial membranes. nsPRP may provide cardioprotection by altering the pathologic electrical activity of the heart, thereby reducing the frequency of arrhythmias. Using optical mapping, we show that LV tissue exposed to global ischemia and treated with nsPRP prior to reperfusion recovered excitability within 1 min in the region injected with nsPRP (but not after the injection of saline). The recovered tissue sustained smoothly propagating waves of excitation, raising the possibility that nsPRP injection can immediately restore (partial) cardiac function in a clinical setting.

nsPRP may also reduce the irritability of the heart and decrease the possibility of arrhythmias. The mechanism involved is currently under investigation. However, arrhythmogenesis has long been associated with the presence and activation of ion channels (Beavis and Garlid 1987). There are many ion channels located in the inner membrane of the mitochondria. The Ca^2+^ uniporter is the primary route for Ca^2+^ entry into the mitochondrial matrix, an event necessary for stimulation of oxidative phosphorylation (Akar et al. 2005; Piot et al. 2008). However, mitochondrial Ca^2+^ overload, as that thought to occur during I/R, can trigger opening of the mPTP. The K^+^ channels (mitoKATP, mitoKCa^2+^, voltage‐activated K^+^) are thought to be cytoprotective (Piot et al. 2008) although there is controversy regarding the specificity of certain K^+^ channel openers and the mechanisms used by these channels to convey protection. There are also channels that are associated with mitochondrial dysfunction. The mPTP channel is thought to be activated by oxidative stress, Ca^2+^, and depolarization. The opening of this channel during I/R (more so during reperfusion) is linked to triggering apoptosis and Ca^2+^ overload, signaling irreversible mitochondrial damage (Lyon et al. 2010). The mitochondrial inner membrane also contains several anion‐selective channels, many with undetermined roles. Of key importance is the IMAC. This channel is reported to mediate the efflux of O^−^_2_ (Brown et al. 2008) and makes it an attractive candidate in the regulation of cellular bioenergetics and redox control (Paky et al. 1993). We speculate that nsPRP may prevent the opening of the mPTP channel through its ability to reduce ROS levels, thereby reducing mitochondrial depolarization and preserving mitochondrial function.

nsPRP contains a wealth of substances that may affect cardiac function. In the studies described in this work, we used an autologous platelet‐rich plasma supernatant formed by activating the platelets to release their proteins and other substances with nanosecond pulsed electric fields. This protein mixture was injected into the myocardium of the heart and no doubt affected cardiac mechanical and electrical performance. For example, ATP possibly released from the dense granules of the platelets may serve as a neurotransmitter or in cell‐to‐cell communication signaling in inflammation and immune responses (Brybsticj 2006). Adenosine may work to precondition the cardiac tissue to the actions of other proteins (Jx and Chen 2015). Glutathione may serve to support the endogenous antioxidant scavenging system so that this system is not overwhelmed by the ROS produced during reperfusion (Salonen et al. 1991).

In summary, we have found that nsPRP enhances left ventricular dp/dt in the Langendorff mouse heart and that its injection into the Langendorff rabbit heart is associated with a faster recovery of electrical activity, therefore reducing the likelihood of arrhythmias. nsPRP also increased the expression of Hsps 27 and 70, with Hsp 70 being necessary for the reduction in ROS levels previously reported. nsPRP also enhances the spare respiratory capacity of mitochondria in the presence and absence of H_2_O_2_, providing the heart with increased cardiac capacity, and making it less vulnerable to bioenergetic exhaustion during I/R. Collectively, these changes function to protect the heart and enhance performance.

## Conflict of Interest

None declared.
